# Fluorescence Lifetime and Intensity of Thioflavin T as Reporters of Different Fibrillation Stages: Insights Obtained from Fluorescence Up-Conversion and Particle Size Distribution Measurements

**DOI:** 10.3390/ijms21176169

**Published:** 2020-08-26

**Authors:** Nataliya R. Rovnyagina, Gleb S. Budylin, Yuri G. Vainer, Tatiana N. Tikhonova, Sergey L. Vasin, Alexander A. Yakovlev, Victor O. Kompanets, Sergey V. Chekalin, Alexander V. Priezzhev, Evgeny A. Shirshin

**Affiliations:** 1 Faculty of Physics, M.V. Lomonosov Moscow State University, Leninskie Gory 1/2, 119991 Moscow, Russia; rovnyagina.nataliya@physics.msu.ru (N.R.R.); avp2@mail.ru (A.V.P.); 2 Institute for Regenerative Medicine, Sechenov First Moscow State Medical University (Sechenov University), 8-2 Trubetskaya Street, 119991 Moscow, Russia; 3 Faculty of Physics, National Research University Higher School of Economics, Myasnitskaya 20, 101000 Moscow, Russia; gleb.budylin@gmail.com (G.S.B.); yvayner@hse.ru (Y.G.V.); 4 Institute of Spectroscopy of the Russian Academy of Sciences, Fizicheskaya, 5, 108840 Moscow, Russia; kompanetsvo@isan.troitsk.ru (V.O.K.); chekalin@isan.troitsk.ru (S.V.C.); 5 International Laser Center of M.V. Lomonosov Moscow State University, Leninskie Gory 1/2, 119991 Moscow, Russia; tikhonova@physics.msu.ru; 6 KDSI Ltd., Gostinickny Proezd 4B, 127106 Moscow, Russia; sergey.vasin@kdsi.ru; 7 Institute of Higher Nervous Activity and Neurophysiology of RAS, Butlerova 5A, 117485 Moscow, Russia; al_yakovlev@rambler.ru

**Keywords:** time-correlated single photon counting (TCSPC), fluorescence up-conversion, dynamic light scattering (DLS), nanoparticle tracking analysis (NTA), fibrils, thioflavin T (ThT)

## Abstract

Thioflavin T (ThT) assay is extensively used for studying fibrillation kinetics in vitro. However, the differences in the time course of ThT fluorescence intensity and lifetime and other physical parameters of the system, such as particle size distribution, raise questions about the correct interpretation of the aggregation kinetics. In this work, we focused on the investigation of the mechanisms, which underlay the difference in sensitivity of ThT fluorescence intensity and lifetime to the formation of protein aggregates during fibrillation by the example of insulin and during binding to globular proteins. The assessment of aggregate sizes and heterogeneity was performed using dynamic light scattering (DLS) and nanoparticle tracking analysis (NTA). Using the sub-nanosecond resolution measurements, it was shown that the ThT lifetime is sensitive to the appearance of as much as a few percent of ThT bound to the high-affinity sites that occur simultaneously with an abrupt increase of the average particle size, particles concentration, and size heterogeneity. The discrepancy between ThT fluorescence intensity and a lifetime can be explained as the consequence of a ThT molecule fraction with ultrafast decay and weak fluorescence. These ThT molecules can only be detected using time-resolved fluorescence measurements in the sub-picosecond time domain. The presence of a bound ThT subpopulation with similar photophysical properties was also demonstrated for globular proteins that were attributed to non-specifically bound ThT molecules with a non-rigid microenvironment.

## 1. Introduction

The process of proteins and peptides aggregation into amyloids has been extensively studied due to its involvement in the pathogenesis of several diseases [1]. Accumulation of amyloid plaques in the human organism is observed for type II diabetes, Alzheimer’s and Parkinson’s disease, and atherosclerosis [2,3,4]. This fact stimulated research of fundamental mechanisms responsible for triggering amyloid formation, intermediates of the fibril formation process, and kinetic aspects of aggregation [5,6]. In the latter case, data can be readily obtained in vitro using a set of methods, including optical techniques (mainly fluorescence spectroscopy of endogenous and exogenous fluorophores, circular dichroism, and light scattering methods), electronic and atomic force microscopy, X-ray scattering, etc. By measuring the kinetics of a particular physical parameter of the system, one may obtain the fibrillation kinetics described by a sigmoid curve in most cases. The conventional approach is to determine several parameters from the aggregation kinetic curve such as the duration of the lag phase and the growth rate and to investigate their behavior upon changing parameters of the system, both molecular environment (ionic strength, temperature, protein concentration, the presence of inhibitory molecules) and molecular structure (e.g., by inducing mutations in the protein structure) in order to study the fibrillation process [5,7,8,9,10,11,12,13,14].

Thioflavin T (ThT) assay is one of the most popular methods to quantify fibrillation kinetics in vitro [15,16]. Fluorescence quantum yield of ThT exhibits three orders of magnitude increase upon specific binding to amyloids [17,18,19], which is due to a rigid fixation of the ThT molecule fragments when incorporated into fibrils. Hence, ThT fluorescence intensity changes can be used as an indicator of fibril formation [19]. Fluorescence analysis is often combined with other methods, such as dynamic light scattering, circular dichroism, etc., to study the aggregation multistage process and establish the fibrillation mechanisms. However, it has been shown that the kinetic change in different physical characteristics obtained using these techniques can vary when studying the same process. In general, asynchronous behavior of fibrillation kinetics, as revealed by ThT fluorescence and other methods, is explained as the consequence of their different sensitivity to certain fibrillation stages and intermediates. Nevertheless, there is currently no systematization and clear explanation of the observed effect in the literature. Thus, one of the methods that most often complement the ThT fluorescence analysis is the dynamic light scattering (DLS). However, when analyzing the experience of using these techniques for monitoring fibrillation, in different works, there is a discrepancy between the dynamics of changes in the ThT fluorescence intensity and in the parameters determined using DLS when analyzing the same system in different works. Some researchers declare that DLS is most sensitive to protein structural rearrangements during fibril formation. At the same time, other works show that the dependence of the ThT fluorescence intensity during aggregation is saturated earlier than the hydrodynamic radius of aggregates determined by DLS [20,21,22]. Moreover, even different photophysical properties of ThT may demonstrate kinetic curves with different parameters (the length of the lag phase, etc.). In the works [23,24,25], asynchronous behavior of ThT fluorescence intensity and lifetime was shown. A higher sensitivity of ThT explained this effect to the formation of protofibrils [23]; however, it was suggested that an alternative explanation could be insufficient time resolution when measuring time-resolved decay of ThT fluorescence [26].

In this work, we focused on investigating the mechanisms that underlay different sensitivity of ThT fluorescence intensity and lifetime to the formation of protein aggregates during fibrillation by the example of insulin. The assessment of aggregation size and heterogeneity was conducted using two complementary methods, DLS and nanoparticle tracking analysis (NTA), and back-to-back comparison of these parameters with ThT’s steady-state and time-resolved fluorescence properties was performed. Fluorescence decay of ThT during fibrillation was studied on a sub-nanosecond and sub-picosecond timescale to determine the impact of ThT subpopulation with ultrafast decay. Although fluorescence spectroscopy with a sub-picosecond time resolution was used to study the photophysical characteristics of the ThT incorporated into mature fibrils [27], analysis of the evolution of ThT ultrafast relaxation along fibrillation was conducted in this work for the first time. We assessed the capabilities of the NTA fluorescence mode to visualize the fibrillation process at late fibrillation stages, a new optical approach for tracking the aggregation kinetics. Collective application of this set of methods revealed a self-consistent picture, which allows for a better understanding of the interconnection between aggregates number and size and ThT fluorescence parameters during fibrillation.

## 2. Results

### 2.1. The Presence of Bound ThT Subpopulation with Ultrafast Relaxation Can Explain Asynchronous Changes of ThT Fluorescence Intensity and Lifetime during Fibrillation

**Figure 1:**
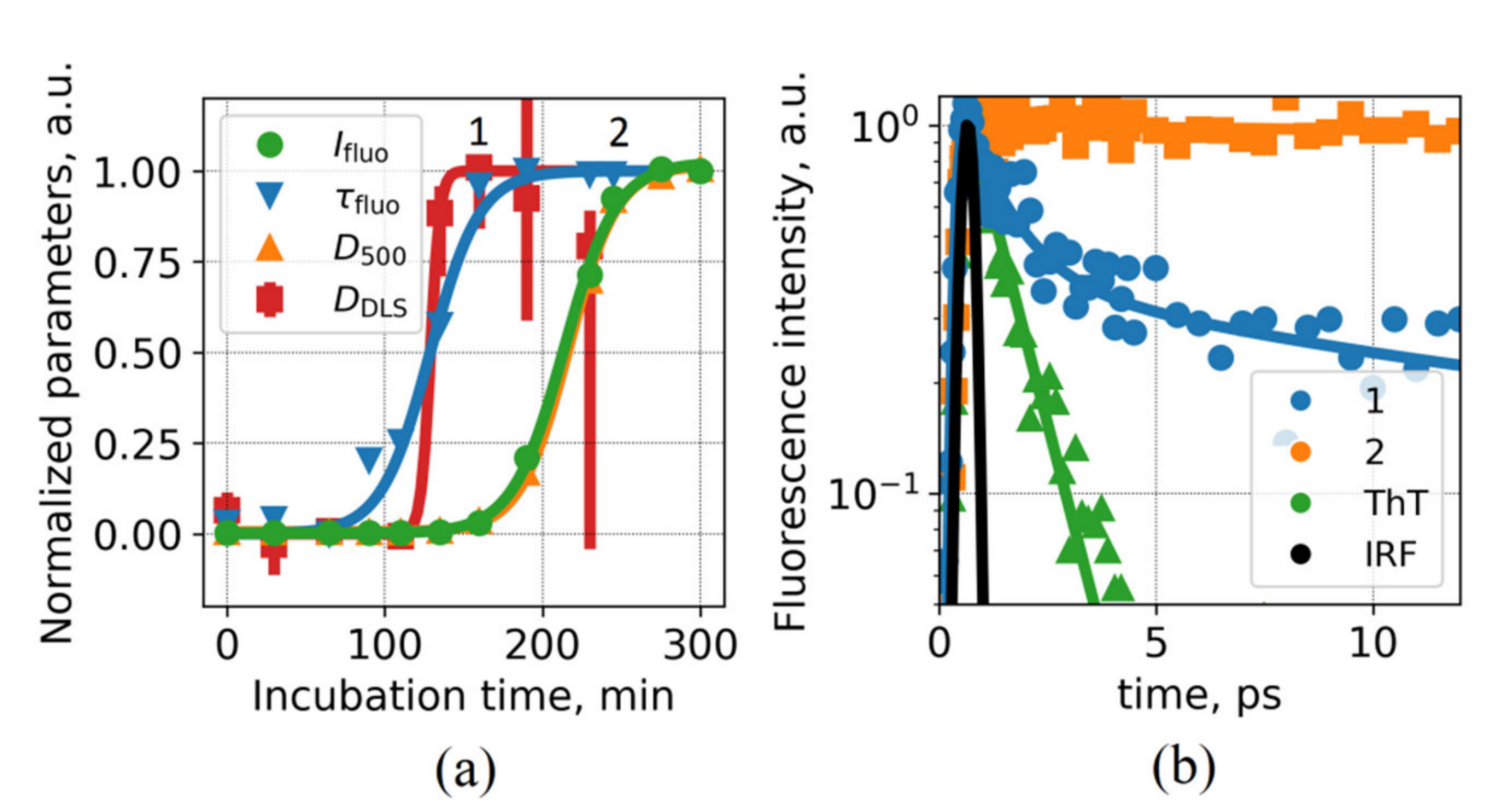
(**a**) The dependence of normalized Thioflavin T (ThT) fluorescence intensity (green) and lifetime (blue) during the fibril formation process of insulin. All curves were normalized to the (0,1) interval. Here, fluorescence lifetime was measured using the time-correlated single photon counting (TCSPC) technique (Figure S1 (Supplementary Materials)) and the absolute values for all the obtained parameters are presented in Table S1 (Supplementary Materials) and in Figure S4 (Supplementary Materials). The orange line corresponds to normalized values of solution turbidity measured at 500 nm (D_500_). The red line corresponds to normalized values of the logarithm of average particle sizes as revealed by dynamic light scattering (DLS). Red bars represent the 25th and 75th percentiles of the particle size distribution measured by DLS. The solid lines represent a sigmoidal fit. Samples with fibrillation times labeled as 1 and 2 in the panel (**a**) were also measured using the up-conversion technique, and corresponding decay curves are presented in the panel (**b**) and labeled as 1 and 2, respectively. The absolute values for all the obtained parameters are presented in Table 1 and Table 2. (**b**) Fluorescence decay curves of ThT in water (green) and in the presence of oligomers and/or protofibrils (blue, point 1 in Figure 1a) and mature fibrils (orange, point 2 in Figure 1a) measured using the fluorescence up-conversion technique. Solid lines correspond to the fitting of the experimental curves to Equation (6). Instrument response function (IRF) is shown with a black line.

Figure 1a demonstrates the kinetics of ThT fluorescence intensity and lifetime during the insulin fibrillation. As can be seen, fluorescence lifetime starts to increase earlier and reaches a plateau when fluorescence intensity enhancement is ~10, compared to ~400 for mature fibrils at the end of the process. Similar behavior of these parameters was reported earlier [23] and was interpreted as a higher sensitivity of ThT fluorescence lifetime to the presence of oligomeric species and/or protofibrils. However, this observation is not consistent with the accepted mechanism of ThT photophysical properties formation according to the molecular rotor principle [28,29].

Namely, ultrafast relaxation of free ThT is due to the formation of the torsional internal charge transfer (TICT) state upon excitation, which results in non-radiative decay on a ~1 ps time scale [28,30,31]. Rigid fixation of ThT, when bound to mature fibrils, prevents this relaxation pathway, leading to a dramatic reduction of the non-radiative decay rate k_nr_ to ~1 ns^−1^ [28]. As a result, both fluorescence intensity F and lifetime τ are increased 1000-fold, as F~τ = (k_r_ + k_nr_)^−1^. Hence, the observed 10-fold increase of ThT fluorescence intensity when bound to fibrils and a simultaneous increase in the ThT fluorescence lifetime up to 2 ns is too small in the scope of the described framework.

This contradiction can be resolved if ThT fluorescence decay is considered on a sub-ps time scale. The importance of “zooming in” the fluorescence decay curves of ThT in fibrils can be deduced from two facts. Firstly, it was demonstrated in [27], that at least two ThT binding sites exist in mature fibrils, and, consequently, ThT molecules bound into different sites may exhibit different photophysical parameters. Despite the observed 1000-fold increase in the intensity and average lifetime of ThT fluorescence, there is a weakly fluorescent subpopulation of bound ThT molecules with ultrafast relaxation on a picosecond time scale. These molecules would then be characterized by a low fluorescence enhancement compared to free ThT, thus lowering the system’s overall fluorescence. Secondly, it was shown in [26], that the similar asynchronous behavior of the ThT photophysical parameters could also be observed for a non-fibrillar system with weakly and strongly fluorescent bound ThT fractions. For that system, numerical modeling was performed, which revealed that when using the TCSPC technique with ~100 ps IRF, even 3% of ThT with ~1 ns lifetime would completely mask the impact of ThT molecules with ~1 ps relaxation. Thus, there is a discrepancy in the ThT optical parameters changes when analyzing such a system. The ThT subpopulation with ultrafast relaxation should be investigated with ~100 fs resolution to avoid contradictions and correctly interpret the data. This hypothesis was verified by measuring fluorescence decay of the ThT-insulin system on different stages of aggregation. Figure 1b reveals that while only a slow decay component was observed for ThT for mature fibrils (point 2 in Figure 1a), fluorescence decay for the prefibrillar stage (point 1 in Figure 1a) exhibited a component with ultrafast decay and >50% amplitude (Table 1). The existence of ThT molecules with ~1 ps lifetime, which could be either free or bound and weakly fluorescent, explains low fluorescence enhancement observed on the prefibrillar stage when ThT fluorescence lifetime saturates.

From the perspective opened up by fluorescence decay measurements with a sub-ps resolution, the emergence of a slow (nanosecond) decay component (as revealed by the TCSPC data) is indicative of the presence of a subpopulation of ThT molecules, incorporated into binding sites with the rigid microenvironment. The time point where fluorescence lifetime reaches saturation could be indicative of a fraction of such molecules of a few percent.

Nevertheless, whether the presence of non-specific ThT binding site with low rigidity and ultrafast ThT fluorescence decay is specific for protofibrils or is a common feature of non-specific interaction of ThT with proteins and protein oligomers remains open. To address this issue, we studied the photophysical characteristics of ThT upon binding to globular proteins.

### 2.2. Ultrafast Fluorescence Decay of ThT Incorporated into Globular Proteins

**Figure 2:**
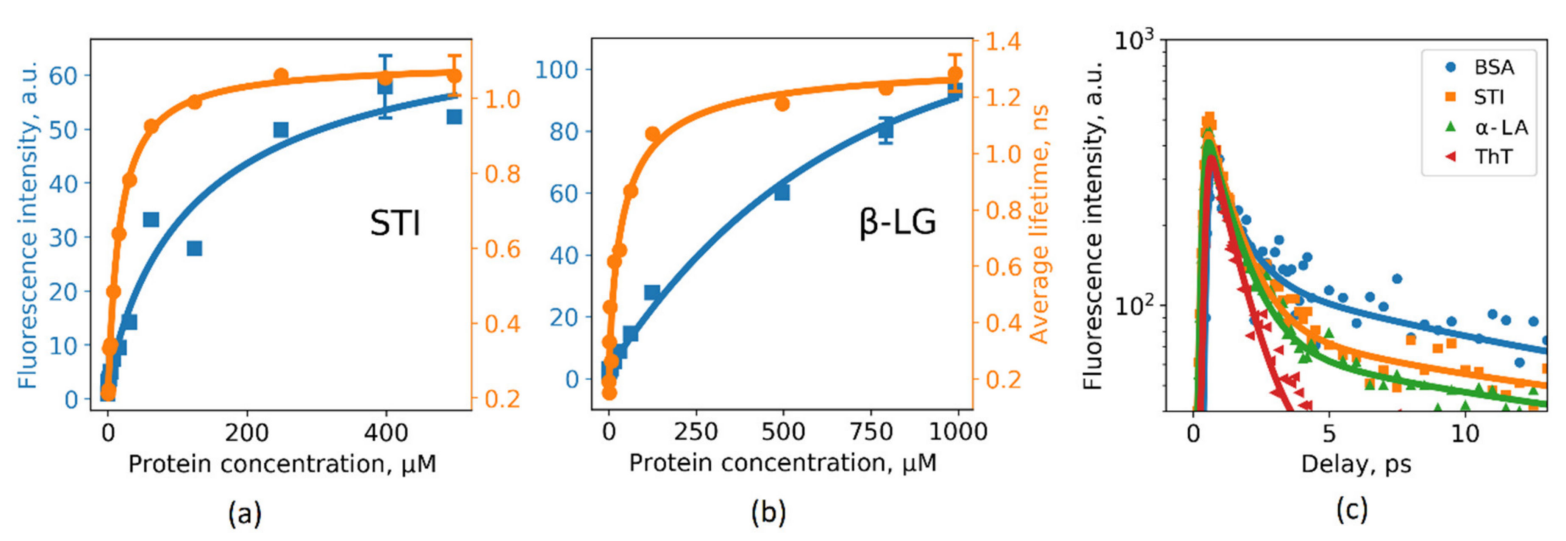
The dependence of ThT fluorescence intensity (**blue**) and lifetime (**yellow**) on the (**a**) STI and (**b**) β-LG concentration. C_ThT_ = 2 µM. Here, fluorescence lifetime was measured using the TCSPC technique. The approximation parameters of the ThT fluorescence decay kinetics are presented in Table 1, in Tables S2 and S3 and in Figure S3 (Supplementary Materials). (**c**) Fluorescence decay curves of ThT in water (**red**) and in the presence of 720 µM α- LA (**green**), 500 µM STI (**yellow**), and 10 mM BSA (**blue**) measured using the fluorescence up-conversion technique. C_ThT_ = 2 µM. Solid lines correspond to the fitting of the experimental curves to Equation (6).

Figure 2 demonstrates the dependence of the ThT photophysical parameters on the concentration of model globular proteins (β-lactoglobulin (β-LG) and soybean trypsin inhibitor (STI)) in the solution. In agreement with the results for other systems, namely, albumins [32], and α-lactalbumin (α-LA) [26], we observed that the ThT fluorescence lifetime saturates at lower protein concentration compared to the ThT fluorescence intensity. This asynchrony was attributed to the presence of a bound ThT subpopulation with ultrafast relaxation [26]. When measuring fluorescence decay with IRF width of σ~100 ps, the true decay kinetics is convolved with IRF. Thus, the observed amplitude of the fast decay component with the lifetime τ is multiplied by the factor ~ exp(x^2^)·erfc(x), where $x\;=\;\sigma /\sqrt{2}\tau$. For τ = 1 ps and σ = 100 ps, this factor corresponds to 0.008. Hence, even a small (>0.008) fraction of specific ThT binding sites with a characteristic fluorescence decay time of ~1 ns can mask the contribution of ThT molecules with ultrafast relaxation to the fluorescence decay.

To verify this suggestion, fluorescence decay of ThT incorporated into α-LA, STI, and BSA was measured with subpicosecond resolution (Figure 2c). ThT and protein concentrations were chosen so that at least 70% of ThT was bound according to the fluorescence titration curves, and the approximation parameters are presented in Table 1. The first component with a lifetime of 1 ps corresponds to the free ThT present in the solution, and to the ThT fraction weakly bound to the protein molecule. The second component with a characteristic lifetime of ~10 ps presumably corresponds to a probe incorporated into the binding sites with a more rigid microenvironment. We also note that a component with a longer decay time (~1 ns) was present in all the studied systems, although it could not be resolved due to the limitations of the setup, thus making its direct comparison to the values obtained using TCSPC impossible.

Summarizing, for all the globular proteins under study, a weak non-specific interaction with ThT was observed (the dissociation constant did not exceed 100 μM), which was accompanied by (i) low fluorescence enhancement (~40) and (ii) the presence of an ultrafast component in fluorescence decay. These two features were also observed for the case of protofibrils, thus suggesting that such photophysical parameters are inherent for weakly bound ThT molecules and cannot be attributed to the binding site’s specific properties.

### 2.3. Particle Size Distribution during Insulin Fibrillation as Revealed from DLS and Its Correlation with ThT Photophysical Parameters

**Figure 3:**
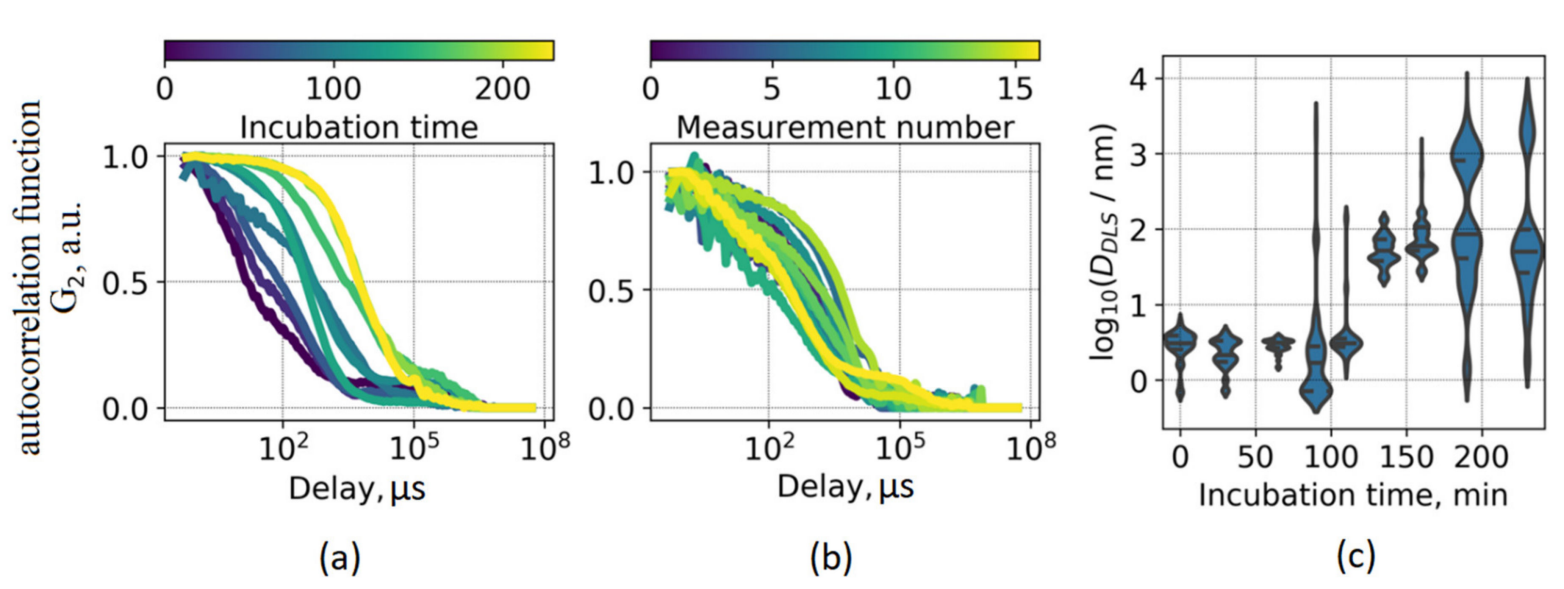
(**a**) Evolution of the averaged correlation functions obtained using DLS during insulin fibrillation. (**b**) Seventeen representative correlation functions were obtained for the same sample (90 min of incubation, see Figure 1a), each measured for 1 s. (**c**). The dependence of the number-averaged particle size distribution on fibrillation time.

To further investigate the interconnection between the ThT photophysical parameters and certain stages of fibrillation, we made use of two complementary diffusion-based techniques (DLS and NTA) to assess particle size distributions at different aggregation times. DLS measurements revealed a gradual shift of scattering fluctuations correlation function (Figure 3a), which is indicative of an increase of the average particle size in the system. However, the direct interpretation of the DLS data for the fibrillation process is complicated due to a high degree of heterogeneity of the system. This fact is illustrated in Figure 3b, where 17 correlation functions for the same sample, each measured during 1 s, are shown. It can be seen that the measurement results vary significantly, and the size distribution is highly polydisperse. Hence, running a single measurement for such a heterogeneous system would result in disguising its complexity.

Figure 3a presents correlation functions averaged over 100 measurements, each performed during 1 s. The processing of each measured correlation function yielded particle size distributions presented in Figure 3c as violin plots. It should be noted that the obtained values of effective sizes of scattering particles cannot be interpreted directly as fibril length due to geometrical differences between prolonged fibrils and the spherical model used for approximation. Nevertheless, these two values should correlate, thus providing insight on changes in protein aggregate dimensions.

The particle sizes varied from ~3 nm at the beginning of the fibrillation process, and the size distribution became multimodal at the later stages, including the emergence of ~100 nm and ~1 µm modes. Importantly, a significant increase in the average particle size occurred at the fibrillation time when ThT lifetime starts to change—this fact is illustrated in Figure 1a, where the average particle size is shown as a function of fibrillation time. At the same time, solution turbidity D_500_ and ThT fluorescence intensity start to increase later (Figure 1a). This trend can be interpreted as follows: (i) on the prefibrillar stage, the formation of a certain number of large (~100 nm) aggregates leads to binding of ~1% of ThT molecules into the sites with rigid microenvironment, resulting in an increase of the ThT fluorescence lifetime, (ii) further formation of larger aggregates leads to the increase of ThT fluorescence intensity until all ThT molecules are bound to rigid sites, and the emergence of more abundant large aggregates leads to an increase of turbidity.

### 2.4. Evolution of Aggregates during Fibrillation as Revealed by the NTA Technique

Simultaneously with the DLS measurements, we used the NTA technique, which is based on the analysis of tracks of single particles detected using their scattering or fluorescence signals. NTA is capable of measuring lower particle concentrations than DLS, as well as of the quantitative determination of particle concentration [33].

**Figure 4:**
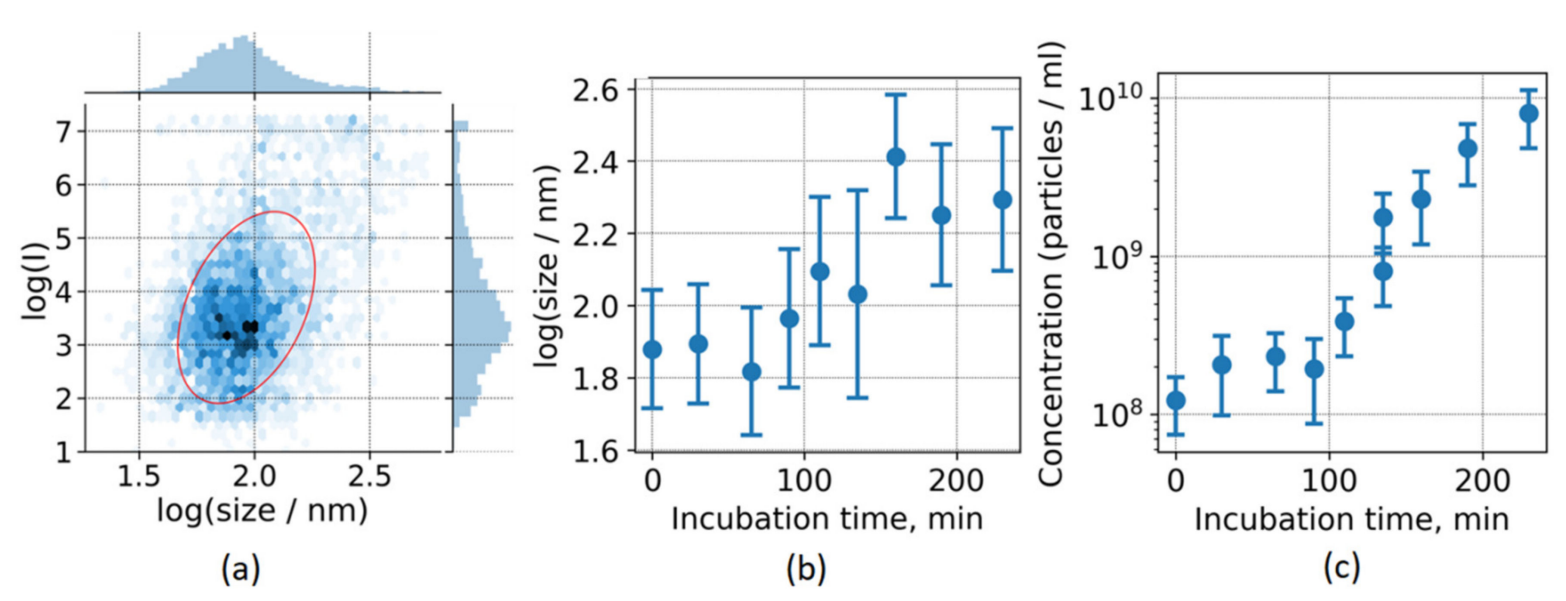
(**a**) Distribution of detected particle intensity and size for 90 min of fibrillation. (**b**) Evolution of the particle size during insulin fibrillation as determined by nanoparticle tracking analysis (NTA). Error bars correspond to the standard deviation obtained using the intensity distribution. (**c**) The dependence of particles concentration on incubation time during insulin fibrillation.

Similarly to the DLS measurements, a high degree of heterogeneity was observed in the NTA experiments. This trend is illustrated in Figure 4a, where the distribution of particle scattering intensity on particle size in the log-log coordinates is shown. It can be seen that the distribution of the observed particles in the intensity-log (size) axes is wide and mostly monomodal with a Gaussian profile. The level corresponding to the fitted Gaussian distribution half-maximum is represented with a red ellipse in Figure 4a. The parameters of the particle size distribution for each incubation time presented using the mean and variance of the log (size) parameter are shown in Figure 4b.

In agreement with the DLS results (Figure 1a and Figure 3c), a significant increase of the median particle size becomes evident simultaneously with an increase of ThT fluorescence lifetime. However, the dynamic range of average particle size revealed by NTA is rather narrow, from ~50 nm to ~250 nm. The lower limit could be caused by the fact that smaller particles cannot be detected due to the sensitivity of the system, while the upper one is most likely connected with the following limitation. The scattering intensity of the particle strongly depends on its size. Thus, to observe the diffusion of small particles, one should increase the sensitivity of the sensor leading sensor saturation for larger particles, errors in the determination of their centroids and overestimation of their diffusive motion.

The NTA method also provides for the concentration of the particles in the solution. As shown in Figure 4c, the concentration of aggregates starts to increase exponentially since 100 min of incubation, i.e., when the ThT lifetime increases. Namely, particle concentration grows from 10^8^ mL^−1^ at the end of the lag phase to 10^10^ mL^−1^, when ThT fluorescence intensity saturates and all ThT is bound.

### 2.5. Assessment of Aggregates Morphology Using the NTA Technique with Fluorescence Detection

Using the fluorescence detection channel of the NTA setup we were able to visualize the process of fibrils formation on the latest stages and to follow alteration of their morphology. Fibrils were stained using the ThT derivative 2M-DMASEBT, which exhibits red-shifted spectral properties.

Similarly, to the case of ThT, when 2M-DMASEBT is incorporated into the β-sheet structure of amyloid fibrils, the torsional movement of molecular fragments toward each other is restricted, and the transition to the non-fluorescent TICT state is blocked [34]. This fixation leads to a significant increase in the fluorescence intensity of the probe upon incorporation into fibrils. The significant difference between 2M-DMASEBT and ThT molecules is the length of the linker between the benzene and benzothiazole rings. The absorption and fluorescence spectra of 2M-DMASEBT aqueous solutions are shifted to the longer wavelength region by approximately 100 nm compared to spectra of ThT aqueous solutions, making the 2M-DMASEBT convenient for application in fluorescence imaging of fibrils using NTA.

**Figure 5:**
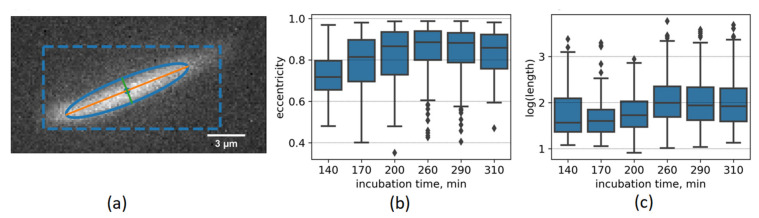
The evolution of fibrils morphology characterized using NTA in the fluorescence mode. (**a**) Image of a fibril labeled with the 2M-DMASEBT dye. The blue dashed rectangle corresponds to the object detected by the algorithm. Blue dot corresponds to the intensity-weighted center of the fibril. Blue ellipse and its axes (yellow and green segments) correspond to a half-maximum level of 2D-Gaussian fit of the region using its covariance matrix, as discussed in the text. (**b**) The dependence of fibril eccentricity distribution on incubation time. Eccentricity is increasing up to 260 min of incubation, demonstrating continuous fibril growth. (**c**) The dependence of fibril length distribution on incubation time.

Only the largest particles with the size of about several microns could be detected in the fluorescence channel. These particles cannot be observed together with smaller (100 nm) particles in the scattering channel at similar detection parameters due to the low concentration (less than one particle per measurement volume) and much higher scattering cross-section leading to detector oversaturation when the particle is in the detection volume. Due to the 2M-DMASEBT bleaching and low detectable particle concentration, the sample was infused into the measurement chamber with the syringe pump. Fibril orientation and elongation can be estimated using the image covariance matrix (Figure 5a). The covariance matrix was used to calculate fibrils’ length, width, and eccentricity as a proxy for fibril elongation.

Figure 5 shows that the distribution of lengths and eccentricities are broad due to a high variety of fibril sizes and their rotation during the observation. The median value of eccentricity distribution is shifted to higher values (increased elongation) during the incubation. Overall, elongation of aggregates proceeds on the latest stages of fibrillation, when both ThT fluorescence and solution turbidity increases (Figure 1).

## 3. Discussion

ThT assay is one of the most popular methods for studying fibrillation kinetics in vitro, and the difference in the time course of ThT fluorescence intensity and other physical parameters of the system raises a question about the applicability of a certain approach for monitoring stages of protein aggregation. In this work, we investigated the asynchronous behavior of ThT fluorescence intensity and lifetime during insulin fibrillation and tried to explain this effect by simultaneously measuring parameters of aggregates by DLS and NTA.

**Figure 6:**
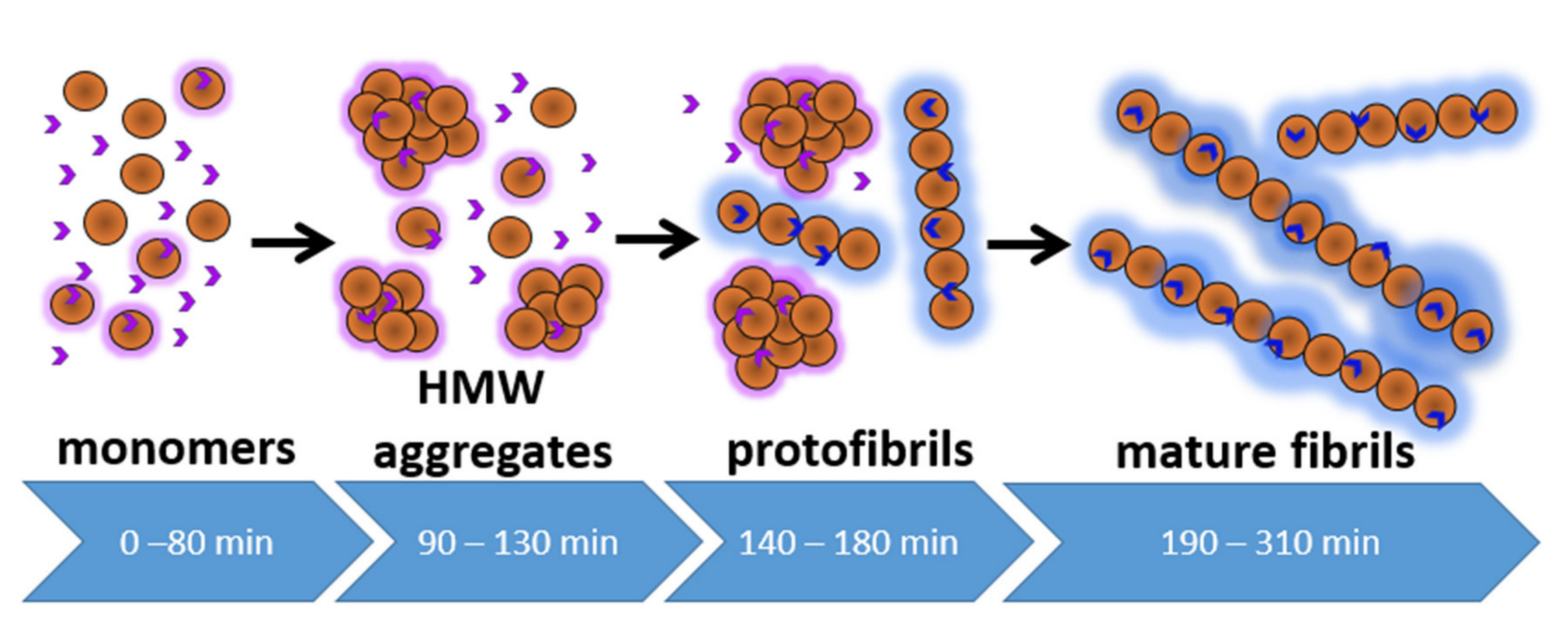
Schematic representation of insulin fibrillation kinetics depicting different stages of aggregation as revealed by ThT fluorescence intensity and lifetime, DLS and NTA. Violet symbols correspond to weakly fluorescent ThT (free or non-specifically bound to protein aggregates); blue symbols correspond to specifically bound ThT with high fluorescence quantum yield. HMW stands for high molecular weight aggregates. The average size of particles in the system starts to increase at stage 2 (90–130 min), ThT fluorescence lifetime saturates at stage 3 (140–180 min), and ThT fluorescence intensity saturates at stage 4 (190–310 min), see Figure 1. Parameters of the stages are presented in Table 2.

The following picture of aggregation processes and its interconnection with ThT fluorescence parameters has been revealed (Figure 6). In the initial stage (up to 80 min), mostly protein monomers are present in the solution (as revealed by DLS), although a low fraction of oligomers can be detected. Namely, NTA revealed the presence of as low as 10^8^ cm^−3^ (≈10^−13^ M) of 60-nm particles in the system (Figure 4 and Table 2). It should be noted that the CONTIN analysis of correlation curves measured using DLS allows obtaining the distribution of the relative number of particles (Figure 1a and Figure 3c) by size, as well as the distribution of the scattered light intensity by the size of the aggregates (Figure S2 (Supplementary Materials)). Due to the highly nonlinear dependence of the scattered light intensity on the particle diameter, the low fraction of high molecular weight oligomers formed before the start of aggregation can be observed using the intensity distributions obtained by the DLS technique. Nevertheless, at the beginning of the lag phase, the concentration of oligomers is much lower than that of monomers (Figure 1a and Figure 3c). According to the literature, the oligomers can be suggested to be amorphous aggregates [35,36]. For instance, in [36], it was suggested that the oligomeric fraction represents pre-assembled protein clusters that act as heterogeneous nucleation centers, without which the rapid formation of ordered aggregates is disrupted.

ThT is weakly fluorescent on this initial stage, with intensity being 3.6-fold higher than for free ThT in aqueous solution (Figure 1). This can be explained by non-specific binding of ThT to monomeric proteins and/or their aggregates, usually accompanied by low fluorescence enhancement [32]. On the next stage (90–130 min), an increase in ThT fluorescence lifetime begins, accompanied by the appearance of a significant amount of aggregates and the increase of size distribution heterogeneity (Figure 3c). Also, an exponential increase in the number of aggregates detected by NTA is observed starting from 90 min of incubation. Between 140 and 160 min of incubation, ThT fluorescence lifetime reaches a plateau of ~2 ns. At the same time, up-conversion measurements of fluorescence decay curves revealed the presence of two components (Figure 1b and Table 1) with ultrafast relaxation (0.95 ps, 67%; 10.6 ps, 22%) and also showed the presence of ThT nanosecond fluorescence lifetime (11%). Due to the time resolution of the TCSPC system, the former fast component is not seen when performing measurements on a sub-ns time scale [26], thus leading to saturation of the determined mean fluorescence intensity of ThT. However, it can be deduced from the ultrafast measurements that the prevailing part of ThT remains weakly fluorescent, i.e., it is not bound into a high-affinity site on fibrils. The presence of slow fluorescence decay can be attributed to the existence of the population of ThT molecules, specifically bound by aggregates and thus characterized by a high fluorescence quantum yield.

Subsequent incubation of the sample leads to a change in ultrafast fluorescence kinetics, namely, a decrease in the amplitude of the thioflavin T subpopulation with a characteristic fluorescence decay time of ~1 ps. The presence of several fractions of a bound ThT probe was previously discussed in the literature based on the results of other methods. In [37], isothermal titration calorimetry (ITC) showed the presence of at least two ThT binding sites in mature insulin fibrils with the first mode characterized by K_d_ of 59 nM and 0.14 mole of ThT per mole of insulin in fibril and the second one with K_d_ of 23 mM and 0.6 moles of bound ThT per mole of insulin in the fibril. A similar observation was made using the equilibrium microdialysis technique to study ThT associated with fibrils from insulin and lysozyme [19,38]. It was assumed that the expected localization of the weaker binding sight was on the surface of the fibrils and that surface binding weakly affected the rotation relaxation time, thus leading only to a slight increase of fluorescence lifetime [27,39]. Hence, the existence of ThT surface binding site in mature fibrils affects its photophysical characteristics. The presence of a weakly fluorescent ThT fraction must be taken into account when analyzing the integral characteristics of ThT during fibril formation. While the studies discussed above demonstrate the existence of the low-affinity binding site in mature fibrils, our study demonstrates the presence of the weakly binding site on the earlier stages of fibril formation, thus complementing the general picture of the process. Further growth of the fibrillar structures leads to an increase of rigid binding site concentration and redistribution of bound ThT from the low-affinity sites to the high-affinity ones. The observed decrease in the contribution of the ThT subpopulation with ultrafast relaxation is also in agreement with the existence of surface binding sites in the amyloid fibrils.

The emergence of rigidly bound ThT subpopulation strongly correlates with the beginning of an abrupt increase of aggregate sizes and concentrations revealed by DLS and NTA (Figure 1a, Figure 3c and Figure 4b). Finally, a 400-fold increase of ThT fluorescence intensity occurs synchronously with an increase in solution turbidity (190–310 min, Figure 1a). This stage is connected with the formation of large particles, which are predominantly mature fibrils rich with beta-sheet structures and high-affinity binding sites for ThT (Figure 3 and Table 2). We also used the fluorescence mode of the NTA setup to assess the properties of these large aggregates, which demonstrate an overall elongation and increase of the eccentricity of particles after 190 min. This fact is also in agreement with the formation of mature fibrils [40].

## 4. Materials and Methods

### 4.1. Samples Preparation

**Scheme 1:**
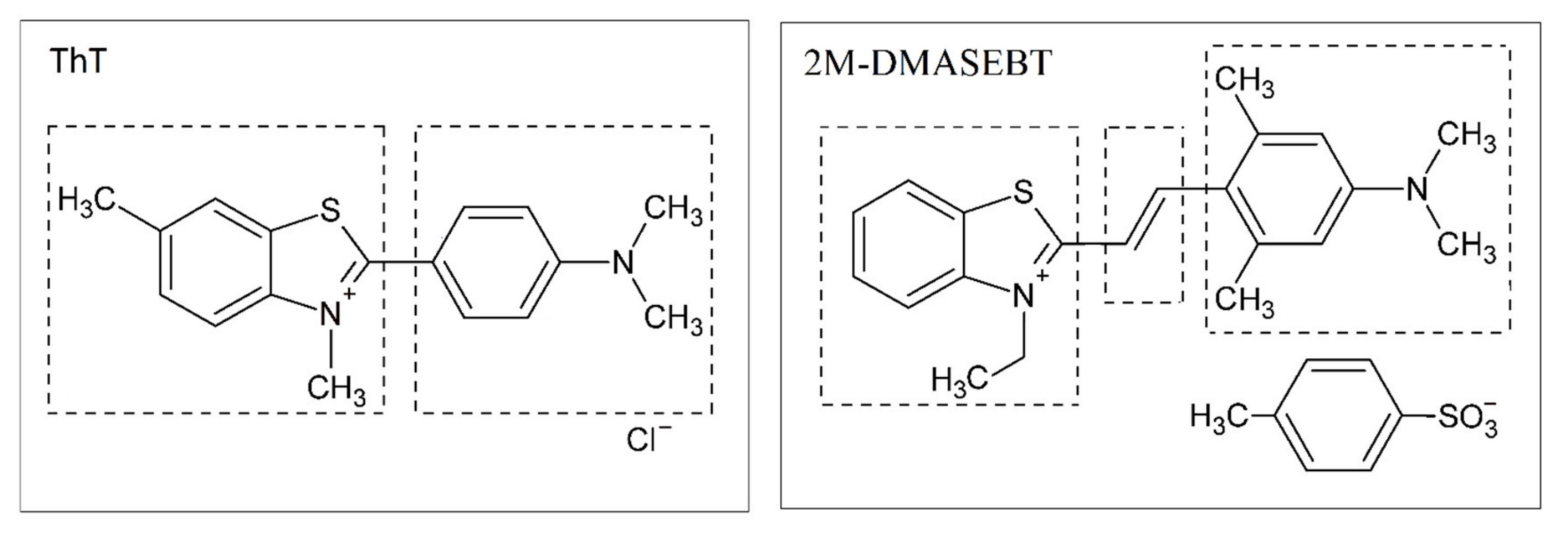
Chemical structure of Thioflavin T and 2M-DMASEBT.

Thioflavin T (ThT) was obtained from “Sigma-Aldrich” (St. Louis, MO, USA) (Scheme 1). Human recombinant insulin (Ins) was purchased from “PanEco” (Moscow, Russia). The final insulin concentration was determined by the absorption at 280 nm using the molar extinction coefficient ε = 5530 M^−1^·cm^−1^ [41].

The buffer for protein solution with pH 2 was prepared by the addition of HCl to 0.1 MNaCl solution. Dry insulin was dissolved in the buffer to a final concentration of 34 µM. The stock insulin solution was filtered before heating using sterile filters with pore size 0.2 nm (Chromafil CA-20/25(S), Macherey-Nagel GmbH, Germany) and stored at −4 °C. To induce fibrillation. the stock solution was incubated at 62 °C and cooled down to room temperature (25 °C) before measurements (steady-state and time-resolved fluorescence measurements using TCSPC, turbidity, up-conversion, DLS, and NTA measurements). To measure the kinetics of fibrillation, aliquots of the stock solution were taken at selected time points. The final ThT concentration was 2 μM and was verified by absorbance at 410 nm using the extinction coefficient ε = 36,000 M^−1^·cm^−1^ [37]. Each aliquot was measured using all the techniques at room temperature.

Bovine serum albumin (BSA) was obtained from MP Biomedicals (Santa Ana, California, USA). Alpha-lactalbumin (α-LA), soybean trypsin inhibitor (STI), beta-Lactoglobulin (β-LG) were obtained from “Sigma-Aldrich” (St. Louis, MO, USA). For up-conversion measurements, dry BSA, α-LA, STI were dissolved in the buffer at the concentration of 10 mM, 720 µM, 500 µM, respectively, at a fixed ThT concentration of 20 μM. For steady-state and time-resolved fluorescence measurements, the stock STI and β-LG solutions were prepared at the concentration of 500 and 1000 µM, respectively, at a fixed ThT concentration of 2 μM and then diluted to perform fluorescence titrations. All measurements were performed in phosphate buffered saline containing 0.01 M phosphate, pH 7.4 at 25 °C.

The nanoparticle tracking analysis experiments with fluorescence detection were carried out with the use of ThT analog 2M-DMASEBT (trans2-(′-(dimethylamino)-2,6-dimethyl styryl)-3-ethyl-1,3- benzothiazole tosylate) (Scheme 1) [42]. The 2M-DMASEBT was used in this experiment because of the red shift of its absorption and fluorescence spectra by approximately 100 nm compared to ThT [43], which allowed to excite it with 488 nm laser used in the NTA setup.

It was previously shown in [44], that the time course of fibril formation might vary under identical initial conditions, particularly when the sample is stirred. In our experiments the duration of the lag phase slightly varied between replicates; however, the trends in the kinetics of the measured parameters remained similar. Hence, we present the time courses of different parameters as obtained in a single experiment, while at least five experiments on the aggregation kinetics were performed.

### 4.2. Steady-State and Time-Resolved Fluorescence Measurements Using TCSPC, Turbidity Measurements

Steady-state fluorescence measurements were performed on a FluoroMax-4 spectrofluorometer (Horiba Jobin-Yvon, Japan-France). ThT fluorescence spectra were obtained at 410 nm excitation (slit width 1 nm) in the 425–660 nm range (slit width 2 nm). The excitation wavelength 410 nm fits the maximum of ThT absorption spectrum [18].

Time-resolved fluorescence measurements of ThT were performed using the time-correlated single photon counting (TCSPC) with the custom-built fluorimeter with the typical setup parameters [25]. The fluorescence was excited with a pulsed 405 nm laser diode (IOS, Saint Petersburg, Russia) delivering 1 pJ and 40 ps FWHM pulses, driven at a repetition rate of 25 MHz. The registration system included a photomultiplier (PMC-100; Becker & Hickl GmbH, Berlin, Germany) and a single photon counter module (SPC-130EM; Becker & Hickl GmbH, Berlin, Germany). The FWHM of the instrument response function (IRF) for this setup was ~200 ps [32].

In case of lifetimes significantly longer than IRF, width kinetics may be fitted with the sum of exponents:

(1) $$F\left(t\right)\;=\;\underset{i}{\sum }a_{i}e^{-\frac{t}{\tau_{i}}}$$

where *a_i_* and τ*_i_* are the pre-exponential factors (amplitudes and lifetimes) for the *i*-th component [45].

Because in our case, some samples demonstrated fluorescence decay times close to IRF duration, the decay curves *F(t)* were fitted by the convolution of a Gaussian function corresponding to the excitation pulse and the sum of exponentials. The convolution of exponential and Gaussian distributions is given by $f\left(t;A_{i},\gamma_{i},\;\sigma ,\;\mu \right)$:

(2) $$\left(t;A_{i},\gamma_{i},\sigma ,\;\mu \right)\;=\;\frac{A_{i}\gamma_{i}}{2}e^{\gamma_{i}\left(\mu -t+\frac{\gamma_{i}\sigma^{2}}{2}\right)}\mathrm{erfc}\left(\frac{\mu +\gamma_{i}\sigma^{2}-t}{\sqrt{2}\sigma }\right)f,$$

(3) $$F\left(t;A_{i},\gamma_{i},\sigma ,\;\mu \right)\;=\;\underset{i}{\sum }f\left(t;A_{i},\gamma_{i},\sigma ,\;\mu \right),$$

where erfc(x) = 1—erf(x) is the complementary error function, μ is the delay corresponding to excitation pulse center, σ is the excitation pulse width, $A_{i}$ is the amplitude of exponentially modified Gaussian distribution corresponding to *i*-th component, $\gamma_{i}$ is the intensity decay rate of *i*-the component. Parameter σ was obtained from the Gaussian fit of the Raman scattering intensity kinetic and fixed for decay curves fitting. Pre-exponential factor $a_{i}$ for decay fitting was calculated as $a_{i}\;=\;A_{i}\gamma_{i}$. Fluorescence lifetimes $\tau_{i}$ were calculated as $\tau_{i}\;=\;1/\gamma_{i}$.

The mean fluorescence lifetime $\tau_{\mathrm{TCSPC}}\;$ was determined as:

(4) $$\tau_{\mathrm{TCSPC}}\;=\;\underset{i}{\sum }\tau_{i}^{2}a_{i}/\underset{i}{\sum }\tau_{i}a_{i}$$

The amplitude-weighted mean fluorescence lifetime was also calculated and yielded similar results; the corresponding data are presented in supporting information.

Solution turbidity D_500_ was measured as optical density at 500 nm using the Lambda-25 spectrophotometer (Perkin-Elmer, Waltham, MA, USA).

### 4.3. Fluorescence Up-Conversion Spectroscopy

Time-resolved fluorescence measurements in the sub-picosecond time domain were carried out using a commercial femtosecond optically gated fluorescence kinetic measurement system FOG100 (CDP Ltd., Moscow, Russia). General principles of the method are reviewed in [46]. The samples were excited by 100-fs pulses at 405 nm (second harmonic of Ti:Sapphire oscillator Mia-Tai, Spectra Physics, Santa Clara, CA, USA). The excitation pulse repetition rate was 80 MHz. The fluorescence collected from the sample was focused on a 0.5-mm β-barium borate (BBO) crystal alongside with the other part of the beam (fundamental, 80 fs, 810 nm) acting as a gate pulse for frequency up-conversion. The gate pulse was delayed by a retroreflector on a stepper-motor controlled delay stage. The upconverted light was focused onto the entrance slit of a double monochromator and detected by a photomultiplier tube. ThT fluorescence emission was collected at the 500 ± 2 nm wavelength to exclude the impact of the Raman scattering of water to the measured signal. The IRF was measured as the temporal profile of the water Raman scattering signal, which is characterized by an ultrafast decay. The reproducibility of the measurements was checked by repeating the experiments in triplicates. The samples were placed in 1 mm rotating cuvette (total volume of 1 mL) to avoid the photo-degradation of the sample.

The decay curves *F*(*t*) were fitted by the convolution of a Gaussian function corresponding to the excitation pulse and the sum of two exponentials (Equation (2)) and a rectangular step corresponding to nanosecond timescale decay. The convolution of exponential and Gaussian distributions is given by $f\left(t;A_{i},\gamma_{i},\;\sigma ,\;\mu \right)$ and convolution of rectangular step with Gaussian function is given by $s\left(t;A_{s},\;\sigma ,\;\mu \right)$:

(5) $$s\left(t;A_{s},\;\sigma ,\;\mu \right)\;=\;A_{s}\left(1+\mathrm{erf}\left(\frac{t-\mu }{\sigma }\right)\right)/2;$$

(6) $$F\left(t;A_{1},A_{2},\gamma_{1},{\;\gamma }_{2},A_{s}\sigma ,\;\mu \right)\;=\;f\left(t;A_{1},\gamma_{1},\sigma ,\;\mu \right)+\;f\left(t;A_{2},\gamma_{2},\sigma ,\;\mu \right)+\;s\left(t;A_{s},\;\sigma ,\;\mu \right),$$

where *A*_s_ is the amplitude of step-function.

The difference between the initial level and the level that the fluorescence decay curve reached at large delays and the nonzero value of the step amplitude may imply that ThT with nanosecond fluorescence lifetime is present in the solution.

### 4.4. Dynamic Light Scattering

Nanoparticle sizes and size distributions were assessed using a Zetasizer Nano S (Malvern Instruments Ltd., Malvern, UK), which employs a 4-mW He–Ne laser operating at a wavelength of 633 nm and a 173° backscatter detector. This technique allowed to measure the time-dependent fluctuations in scattering intensity and to calculate the size of particles within the sample. The correlation function of the signal decays (multi)exponentially with a rate dependent on the diffusion of the particles being measured. General principles of the method are reviewed in [47]. The intensity-weighted size distributions were obtained from the analysis of the correlation functions using the Multiple Narrow Modes algorithm in the instrument software.

All measurements in this study were performed at a temperature of 25 °C. Samples were placed in disposable cuvettes with a 1-cm optical path. After thermal equilibration of the samples to room temperature (typically about 5 min), 100 autocorrelation functions measured for 1 s were collected for each sample.

### 4.5. Nanoparticle Tracking Analysis (NTA)

NTA measurements were performed with the Nanosight NS300 (Malvern Instruments Ltd., Malvern, UK) equipped with a 485-nm laser and a CCD camera. With the use of a high-resolution camera, the trajectories of the particles scattering laser radiation were obtained. The NTA 3.0 software was used for capturing video and data analysis. The trajectories were analyzed and the diffusion coefficient, hydrodynamic radius, and particle concentration were obtained using built-in software [33]. Video tracking was performed for 30 s time intervals with at least 5 measurements for each sample. The sample chamber was visually checked to avoid the presence of air bubbles. The NanoSight pump was set to 0 (no flow) and to 25 a.u. in the case of fluorescence measurements.

In the case of the fibrils investigation in the presence of the fluorescent probe, the signal intensity of the observed objects was substantially lower than the scattering signal for the objects with the size of few nanometers. Also, the fluorescent objects became detectable by CCD only at quite substantial sizes, i.e., a few microns. Thus, the built-in software developed to process the Brownian motion of nanoparticles was unable to perform adequate analysis of micro-objects. The fibril sizes, orientation, and eccentricity were estimated using the image moments. Due to a high number of frames in the detected video, the fibril image selection and its size characterization were automated with the script. A detailed description of the algorithm is given in the supporting information.

## 5. Conclusions

Summarizing, the sensitivity of different optical parameters to the formation of aggregates during insulin fibrillation was assessed. It was shown that ThT lifetime is sensitive to the appearance of as much as a few percent of ThT bound to high-affinity sites. Saturation of the ThT fluorescence lifetime happens simultaneously with an abrupt increase of average particle size, particle concentration, and sizes heterogeneity. The parameters, which change at later stages, are ThT fluorescence intensity and turbidity of the solution. The discrepancy between ThT fluorescence intensity and a fluorescence lifetime determined by TCSPC can be explained as the consequence of the presence of a ThT molecules fraction with ultrafast decay and weak fluorescence that can be revealed using the up-conversion technique. The presence of these molecules is a common feature of the ThT non-specific interaction with proteins and protein oligomers. The impact of these molecules is not detectable when performing measurements on a sub-nanosecond time scale. Overall, all the methods are indicative of the same process—formation of higher-order aggregates but exhibit different behavior of kinetic curves due to sensitivity and saturation limit. For instance, if ThT fluorescence lifetime was measured with the sub-picosecond resolution using fluorescence up-conversion spectroscopy, it would change synchronously with fluorescence intensity, as both parameters are related to changes in the non-radiative decay rate. Analogously, earlier increase and saturation of DLS parameters compared to solution turbidity are connected to the different sensitivity of these methods to the presence of aggregates, while both of them measure aggregate formation. According to these, one may indeed detect earlier or later stages of aggregation by choosing a method with higher or lower sensitivity, respectively.

## Figures and Tables

**Table 1:** Comparison of ThT photophysical parameters when bound to proteins and insulin fibrils (average lifetime measured using TCSPC (τTCSPC), enhancement factor (EF), and the approximation parameters of the ThT fluorescence decay kinetics measured using fluorescence up-conversion (t1 was fixed at a value of free ThT fluorescence lifetime). Enhancement factor (EF) is the ratio of ThT fluorescence intensity in fibrils to that in aqueous solution.

Protein	EF	τ _TCSPC_, ns	Fluorescence Up-Conversion		
			t_1_, ps	t_2_, ps	a_1_/a_2_
Free ThT	-	0.20 ± 0.05	0.95	-	-
α-LA	34	2.80 ± 0.15	0.95	11.9 ± 2.6	9.2 ± 1.7
β-LG	93	2.57 ± 0.13	-	-	-
STI	52	2.58 ± 0.13	0.95	11.2 ± 2.1	8.5 ± 1.1
BSA	35	1.12 ± 0.06	0.95	12.3 ± 4.1	2.8 ± 0.5
Protofibrils (160 min)	9.4	1.77 ± 0.09	0.95	10.6 ± 2.4	2.8 ± 0.8
Mature fibrils (245 min)	415	1.80 ± 0.08	315 ± 15	-	-

**Table 2:** The parameters of the ThT-insulin fibrils system measured for different stages of aggregation (Figure 6).

Observation Time, min.	0	90	160	245
τ _TCSPC_, ns	0.85 ± 0.03	1.02 ± 0.05	1.77 ± 0.09	1.80 ± 0.08
Normalized fluorescence intensity	1	3.6	9.4	415
Turbidity, a.u.	1	38	130	4900
log(D_DLS_/nm)	0.50 ± 0.13	0.28 ± 0.35	1.77 ± 0.13	1.69 ± 0.30
log(D_NTA_/nm)	1.89 ± 0.16	1.96 ± 0.19	2.4 ± 0.17	2.29 ± 0.20

## Supplementary Materials

Supplementary Materials can be found at https://www.mdpi.com/1422-0067/21/17/6169/s1.

## Abbreviations

TCSPC	time-correlated single photon counting
DLS	dynamic light scattering
NTA	nanoparticle tracking analysis
ThT	thioflavin T
BSA	bovine serum albumin
β-LG	β-lactoglobulin
α-LA	*α*-lactalbumin
STI	soybean trypsin inhibitor
