# Supplementary material for: Fluorescence Lifetime and Intensity of Thioflavin T as Reporters of Different Fibrillation Stages: Insights Obtained from Fluorescence Up-Conversion and Particle Size Distribution Measurements
Source: Int J Mol Sci. 2020 Aug 26;21(17):6169. doi: 10.3390/ijms21176169 (PMC7504639; doi:10.3390/ijms21176169)
Supplement: Supplementary file 1 [file ijms-21-06169-s001.pdf]

Supplementary material

Fluorescent NTA images characterization.

The algorithm was implemented using the Python programming language and included two steps: fibril detection in the image and characterization of fibril image using image moments.

Detection step.

Each video frame was gaussian blurred with the kernel size of 9x9 using cv2 library, and after that the boolean image was created using the pixels with intensity higher than the threshold, which was chosen as the median intensity of a frame plus fixed value (3 a.u. in our case). Next, the morphological closing of the result was used for image segmentation. Segmentation was performed using the "label" function of the skimage measure module. Thus, every image was segmented into regions characterized by a weighted centroid, bounding box and weighted central moments. Next, frames' segments were joined into a moving fibril object using the trackpy library with a position predictor using the most recent velocity. As a result, false object detection was discarded and it became possible to estimate object properties using statistics over the time of its presence in the video.

Characterization step.

Each detected fibril image was characterized using weighted central moments. The image covariance matrix was constructed using central moments. After that, fibril orientation, elongation and eccentricity were estimated using its eigenvalues and eigen vectors.

Table S1. The fitting parameters of the ThT fluorescence decay kinetics during fibrillation using TCSPC. The average lifetime was calculated as $\boldsymbol{\tau'}_{TCSPC} = (a_1t_1+a_2t_2)/(a_1+a_2)$, $\boldsymbol{\tau}_{TCSPC} = (a_1t_1^2+a_2t_2^2)/(a_1t_1+a_2t_2)$.

Fibril incubation time, min	a_1, %	t_1, ns	a_2, %	t_2, ns	τ'_{TCSPC}, ns	τ_{TCSPC}, ns
**0**	95	0.24	5	2.15	0.34	0.85
**30**	94	0.25	6	2.00	0.36	0.86
**65**	95	0.22	5	2.11	0.30	0.82
**90**	94	0.30	6	2.38	0.43	1.02
**110**	89	0.20	11	1.83	0.38	1.07
**135**	82	0.32	18	2.11	0.65	1.39
**160**	52	0.75	48	2.15	1.42	1.77
**190**	91	1.45	9	3.40	1.62	1.81
**230**	98	1.60	2	5.46	1.66	1.80
**245**	99	1.60	1	5.71	1.66	1.80

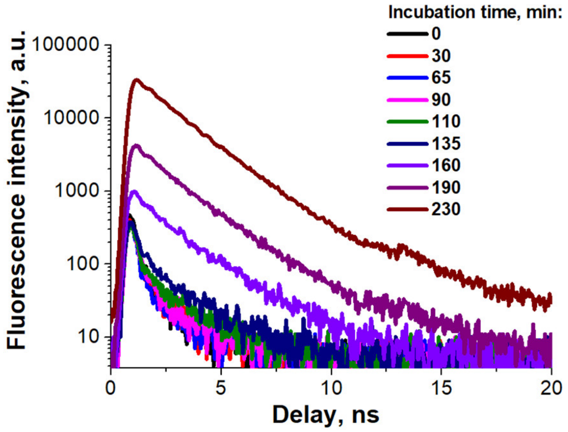

Figure S1. Fluorescence decay curves of ThT during the fibril formation process measured using the TCSPC.

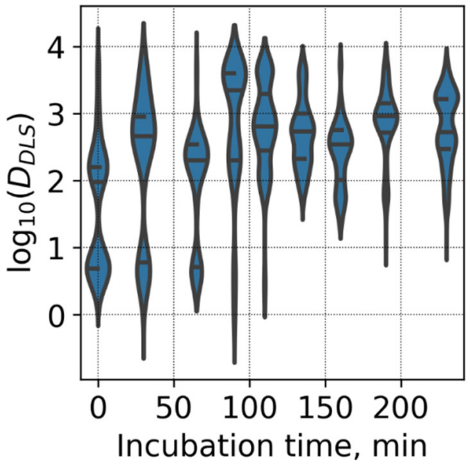

Figure S2. The dependence of the particle size distribution on fibrillation time. Note that each distribution represents dependence of scattering particle number (not intensity) on the particle size. The distribution of the scattered light intensity by the aggregates size was obtained using the CONTIN analysis of intensity correlation functions measured using DLS technique during insulin fibrillation. Size distribution was calculated using the intensity distribution.

Table S2. The fitting parameters of fluorescence decay kinetics measured using TCSPC for the ThT-STI system. The average lifetime was calculated as $\tau'_{TCSPC} = (a_1t_1+a_2t_2+a_3t_3)/(a_1+a_2+a_3)$, $\boldsymbol{\tau}_{TCSPC} = (a_1t_1^2+a_2t_2^2+a_3t_3^2)/(a_1t_1+a_2t_2+a_3t_3)$.

[STI], μM	a_1, %	t_1, ns	a_2, %	t_2, ns	a_3, %	t_3, ns	τ'_{TCSPC}, ns	τ_{TCSPC}, ns
**498**	62	0.38	35	1.76	3	7.41	1.06	2.58
**398.4**	62	0.37	35	1.77	3	7.44	1.05	2.59
**249**	63	0.37	35	1.8	2	7.61	1.06	2.62
**124.5**	64	0.34	33	1.7	3	7.05	0.99	2.53
**62.25**	65	0.3	32	1.66	3	7.02	0.92	2.53
**31.13**	68	0.23	29	1.51	3	6.33	0.78	2.40
**15.6**	73	0.18	24	1.42	3	6.07	0.64	2.30
**7.8**	79	0.13	19	1.38	2	6	0.48	2.23
**3.9**	84	0.1	14	1.3	2	5.33	0.34	1.99
**1.95**	86	0.11	12	1.31	2	5.74	0.33	1.96
**0.97**	91	0.08	8	1.18	1	5.17	0.22	1.82
**0.48**	92	0.08	7	1.22	1	5.5	0.21	1.77

Table S3. The fitting parameters of fluorescence decay kinetics measured using TCSPC for the ThT-β-LG system. Average lifetime was measured as $\tau'_{TCSPC} = (a_1t_1+a_2t_2+a_3t_3)/(a_1+a_2+a_3)$, $\boldsymbol{\tau}_{TCSPC} = (a_1t_1^2+a_2t_2^2+a_3t_3^2)/(a_1t_1+a_2t_2+a_3t_3)$.

[β-LG], μM	a_1, %	t_1, ns	a_2, %	t_2, ns	a_3, %	t_3, ns	τ'_{TCSPC}, ns	τ_{TCSPC}, ns
**991**	56	0.57	43	1.91	1	10.46	1.28	2.57
**792.8**	51	0.5	47	1.74	2	8.4	1.23	2.36
**495.5**	51	0.46	47	1.67	2	7.77	1.18	2.22
**124**	51	0.38	46	1.56	3	6.67	1.07	2.06
**61.9**	55	0.24	42	1.36	3	5.34	0.87	1.93
**30.95**	66	0.18	31	1.32	3	5.02	0.66	1.84
**15.5**	72	0.2	25	1.36	3	5.14	0.62	1.88
**7.74**	86	0.08	13	0.97	1	4.22	0.26	1.63
**3.9**	83	0.18	16	1.43	1	7.06	0.45	2.08
**1.9**	84	0.12	14	1.06	2	4.61	0.33	1.68
**0.97**	92	0.05	7	0.88	1	4.24	0.15	1.59
**0.49**	91	0.07	8	1.01	1	5.11	0.19	1.69

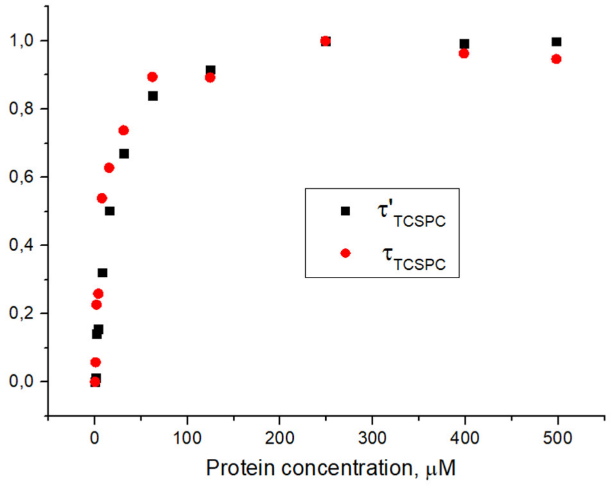

Figure S3. The dependence of ThT average fluorescence lifetime on STI concentration. C_{ThT} = 2 μM. Here, fluorescence lifetime was measured using the TCSPC technique. $\tau'_{TCSPC} = (a_1t_1+a_2t_2+a_3t_3)/(a_1+a_2+a_3)$, $\boldsymbol{\tau}_{TCSPC} = (a_1t_1^2+a_2t_2^2+a_3t_3^2)/(a_1t_1+a_2t_2+a_3t_3)$.

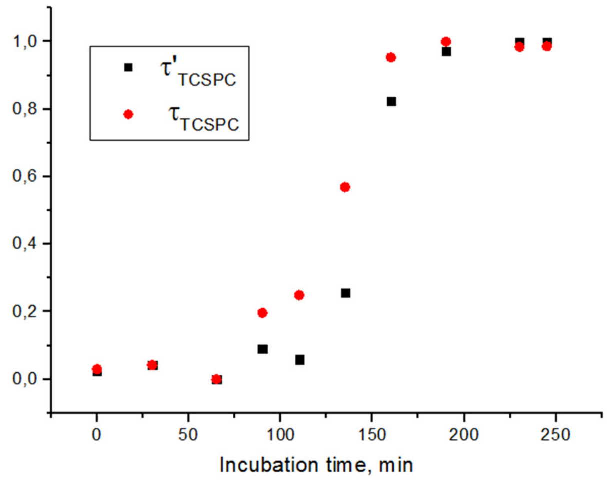

Figure S4. The dependence of ThT average fluorescence lifetime during the fibril formation process. C_{ThT} = 2 μM. Here, fluorescence lifetime was measured using the TCSPC technique. $\boldsymbol{\tau'}_{TCSPC} = (a_1t_1+a_2t_2)/(a_1+a_2)$, $\boldsymbol{\tau}_{TCSPC} = (a_1t_1^2+a_2t_2^2)/(a_1t_1+a_2t_2)$.
